# Weekly high-dose cisplatin is a feasible treatment option: analysis on prognostic factors for toxicity in 400 patients

**DOI:** 10.1038/sj.bjc.6600884

**Published:** 2003-04-15

**Authors:** F E de Jongh, R N van Veen, S J Veltman, R de Wit, M E L van der Burg, M J van den Bent, ASTh Planting, W J Graveland, G Stoter, J Verweij

**Affiliations:** 1 Department of Medical Oncology, Daniel den Hoed Cancer Center, Erasmus University Medical Center Rotterdam, PO Box 5201, 3008 AE Rotterdam, The Netherlands; 2 Department of Neuro-Oncology, Daniel den Hoed Cancer Center, Erasmus University Medical Center Rotterdam, 3008 AE Rotterdam, The Netherlands; 3 Department of Biostatistics, Daniel den Hoed Cancer Center, Erasmus University Medical Center Rotterdam, 3008 AE Rotterdam, The Netherlands

**Keywords:** cisplatin, chemotherapy, toxicity, prognostic factors

## Abstract

In the present study we describe the toxicity of weekly high-dose (70–85 mg m^−2^) cisplatin in 400 patients (203 men, 197 women; median age 54 years) with advanced solid tumours treated in the period 1990–2001 who took part in phase I/II trials, investigating the feasibility and efficacy of weekly cisplatin alone, or in combination with paclitaxel or etoposide. Cisplatin was administered in 250 ml NaCl 3% over 3 h, for six intended administrations. The mean number of administrations was 5.3 (range, 1–6 administrations). Reasons not to complete six cycles were disease progression (7.5%), haematological toxicity (9%), nephrotoxicity (7%), ototoxicity (2.5%), neurotoxicity (1%), gastrointestinal toxicity (1%), cardiovascular complications (0.5%) or a combination of reasons including noncompliance and patient's request (5.5%). Logistic regression analysis was used to evaluate baseline parameters for prognostic value regarding toxicity. Leukopenia correlated with etoposide cotreatment, and thrombocytopenia with cisplatin dose and prior (platinum-based) chemotherapy. Risk factors for nephrotoxicity were older age, female gender, smoking, hypoalbuminaemia and paclitaxel coadministration. Neurotoxicity >grade 1 (11% of patients) was associated with prior chemotherapy and paclitaxel coadministration. Symptomatic hearing loss occurred in 15% with anaemia as the predisposing factor. We conclude that *w*eekly high-dose cisplatin administered in hypertonic saline is a feasible treatment regimen.

*CIS*-Diamminedichloroplatinum (cisplatin) is a commonly used cytotoxic agent with a broad spectrum of activity against solid malignant tumours, including germ cell, ovarian, endometrial, cervical, urothelial, head/neck and lung cancer. When cisplatin was first approved for commercial use in 1978, the major toxicities were severe nausea and vomiting and a high incidence of renal dysfunction. Although these adverse effects are still of concern, they can be significantly reduced by the use of 5HT_3_-receptor antagonists and vigorous hydration (Pinzani *et al*, 1994). Administration of cisplatin in hypertonic saline may further alleviate nephrotoxic side effects (Ozols *et al*, 1984). Protective measures against nausea, vomiting and renal dysfunction have created the opportunity to increase the (individual and cumulative) cisplatin dose. With mild to moderate myelosuppression during conventional 3- or 4-weekly therapy, neurotoxicity (Cersosimo, 1989) and ototoxicity (Laurell and Jungnelius, 1990) have emerged as the remaining major dose-limiting side effects.

The rationale for weekly administration of high-dose cisplatin is based on the tumour biological principle that frequent administration of chemotherapy in a high dose results in more effective killing of cancer cells and potentially reduces the risk of developing chemotherapy resistance. Furthermore, by shortening the treatment interval, tumour cells have less time for regrowth between treatment courses. Weekly administration of cisplatin has extensively been studied at our institution in a range of prospective clinical trials (Planting *et al*, 1993,^1994^,1995a,1995b,1997a,1997b; van der Burg *et al*, 1998; van den Bent *et al*, 1999,^2002^).

In the present analysis, we have pooled data of 400 patients treated with cisplatin at weekly doses of 70–85 mg m^−2^ for an intended number of six administrations, with the goal of describing in detail the toxicity of this weekly regimen and of identifing predisposing factors for the development of side effects, with an emphasis on nephrotoxicity, neurotoxicity and ototoxicity.

## PATIENTS AND METHODS

### Patient selection

Patients who had been treated with weekly high-dose cisplatin in the period 1990–2001 were analysed. The majority of patients participated in phase I/II clinical trials (Planting *et al*, 1993,^1994^,1995a,1995b,1997a,1997b; van der Burg *et al*, 1998,^2002^; van den Bent *et al*, 1999). All study protocols were approved by the Institutional Ethics Board and all participating patients gave written informed consent. According to the inclusion criteria of the trials, patients were required to have locally advanced or metastatic cancer with no better treatment options than weekly cisplatin as a single agent or in combination with either i.v. paclitaxel or oral etoposide. Age had to be ⩾18 years, WHO performance status 0–2, and life expectancy more than 12 weeks with adequate haematopoietic, renal and hepatic function at study entry. Based on favourable treatment results (Planting *et al*, 1997b), patients with locally advanced head/neck cancer could be offered treatment with weekly cisplatin induction chemotherapy followed by radiotherapy outside study protocols.

### Treatment

Cisplatin was administered at a dose of 70–85 mg m^−2^ on days 1, 8, 15, 22, 29 and 36 as a single agent, or at a dose of 70 mg m^−2^ on days 1, 8, 15, 29, 36 and 43 in combination with oral etoposide or i.v. paclitaxel. Cisplatin powder was dissolved in 250 ml NaCl 3% and administered by i.v. infusion over 3 h. Patients received prehydration with 1 l normal saline or dextrose-saline and posthydration with 3 l normal saline or dextrose-saline supplemented with KCl (20 mmol l^−1^) and MgSO_4_ (2 g l^−1^). Antiemetic prophylaxis consisted of a 5HT_3_ antagonist in combination with dexamethasone. Diuretics were not administered routinely.

### Dose intensity

Dose reductions were not allowed. Cisplatin single-agent treatment was postponed 1 week for a maximum of 3 weeks if WBC <2.5 × 10^9^ l^−1^ and/or platelets <75 × 10^9^ l^−1^. When used in combination with etoposide, cisplatin administration was postponed in the case of WBC <2.5 × 10^9^ l^−1^ and/or platelets <75 × 10^9^ l^−1^ on day 8 or day 36, WBC <1.5 × 10^9^ l^−1^ and/or platelets <50 × 10^9^ l^−1^ on day 15 or day 43, and WBC <3.0 × 10^9^ l^−1^ and/or platelets <100 × 10^9^ l^−1^ on day 29. With the cisplatin/paclitaxel regimen, treatment was postponed if WBC <1.0 × 10^9^ l^−1^ and/or platelets <50 × 10^9^ l^−1^ on days 8, 15, 36 or 43, and if on day 29 WBC <3.0 × 10^9^ l^−1^ and/or platelets <100 × 10^9^ l^−1^. Cisplatin was discontinued if creatinine clearance fell below 45 ml min^−1^ or in case of neurotoxicity >grade 2.

The planned dose intensity was calculated by dividing the planned total dose of cisplatin (mg m^−2^) by the planned duration of treatment given in weeks (i.e. 6 weeks for cisplatin single agent and 7 weeks for cisplatin in combination with etoposide or paclitaxel). The achieved dose intensity was calculated by dividing the total administered dose (mg m^−2^) by the actual treatment duration given in weeks. Patients who did not complete treatment due to nontoxicity reasons (e.g. disease progression, noncompliance) were not evaluated for achieved dose intensity.

### Data collection and statistical analysis

Pretreatment and follow-up studies have been reported in detail elsewhere (Planting *et al*, 1993,^1994^,1995a,1995b,1997a,1997b; van der Burg *et al*, 1998,^2002^; van den Bent *et al*, 1999). Patients' baseline characteristics analysed included age, sex, length, weight, body-surface area (BSA), blood pressure, smoking and drinking habits, amount of weight loss, performance status, tumour type, prior anticancer treatment, planned cisplatin dose and dose intensity, and cytotoxic comedication (etoposide, paclitaxel or none). Physical examination, laboratory tests and assessment of toxicity were performed weekly during treatment. Laboratory tests included haemoglobin, WBC, granulocytes, platelets, albumin, bilirubin, alkaline phosphatase, alanine aminotransferase (ALT), aspartate aminotransferase (AST), lactate dehydrogenase (LDH), sodium, potassium, calcium, magnesium, creatinine and creatinine clearance measured by 24-h urine collection. In addition, creatinine clearance was estimated using the Cockroft and Gault equation. Toxicity was assessed according to the Common Toxicity Criteria (CTC), version 1.0, National Cancer Institute (NCI), and, for the present analysis, was evaluated after three administrations of weekly cisplatin, after six administrations and 3 months after completion of the weekly cisplatin regimen. Since audiograms were not routinely performed, grade 1 ototoxicity was not reported. Patients who went off treatment before the fifth administration of cisplatin for reasons other than the evaluated toxicity were excluded from analysis of that toxicity, in order to prevent under-reporting of toxicity.

Logistic regression analysis was used in order to test baseline parameters for their prognostic value regarding toxicity. Patients with neurological symptoms or hearing impairment at baseline were excluded from logistic regression analysis for neurotoxicity and ototoxicity, respectively. In order to eliminate the influence of baseline serum creatinine and creatinine clearance on renal toxicity assessment, renal toxicity was defined as a ⩾25% decline in the estimated creatinine clearance from baseline. After univariate analysis, all baseline parameters were presented to the multivariate model that used a stepwise procedure starting with an empty model and putting the most significant factor at that time into the model. This process was repeated until the *P*-value of the factor involved exceeded 0.025. This level of statistical significance was chosen to reduce the risk of finding purely coincidental associations in view of the large amount of factors analysed. *P*-values were calculated using the Wald test. For each parameter remaining in the multivariate model, an odds ratio (OR) with 95% confidence interval (CI) for the development of toxicity was calculated.

## RESULTS

### Patient characteristics

Patient characteristics are shown in Table 1

**Table 1:** Patient characteristics (*n*=400)

	**No. of patients**	**(%)**
*Sex*		
Male	203	51
Female	197	49
		
*Age (years)*		
Median	54	
Range	19–79	
		
*WHO performance status*		
0	157	39
1	206	51
2	34	9
Unknown	3	1
		
*Tumour type*		
Head and neck cancer	155	39
Ovarian cancer	108	27
CUP	47	12
NSCLC	36	9
Mesothelioma	24	6
Glioma	18	4
Miscellaneous	12	3
		
*Prior chemotherapy*		
None	308	77
Platinum-based	88	22
Nonplatinum-based	4	1

WHO=World Health Organization; CUP=Carcinoma with unknown primary; NSCLC=nonsmall cell lung cancer.

. A total of 400 patients (203 males, 197 females) who had been receiving a total of 2116 weekly cisplatin administrations were included in the study. Predominant tumour types were head and neck cancer (39%) and ovarian cancer (27%). Of the 92 patients with prior chemotherapy, 88 patients had recurrent ovarian cancer after one or more platinum-based regimens (cisplatin pretreatment in 40 patients).

The planned cisplatin dose was 70 mg m^−2^ for 323 patients (81%) and 80 mg m^−2^ for 70 patients (18%). Five patients received 75 mg m^−2^, and two received 85 mg m^−2^. Cisplatin was administered as a single agent to 143 patients (36%); 196 patients (49%) received cisplatin in combination with oral etoposide and 61 (15%) with i.v. paclitaxel. A total of 263 patients (66%) received six cisplatin administrations: 151 without any delay (38%), 64 with 1 week delay (16%), 39 with 2 weeks delay (10%), seven with 3 weeks delay (2%), and two with 4 weeks delay (0.5%). From the patients that received four (*n*=30) or five (*n*=59) administrations of cisplatin, 55 had no treatment delay (14%), 18 had 1 week delay (4.5%), 10 had 2 weeks delay (2.5%) and six had 3 weeks delay (1.5%); from the patients that received one (*n*=8), two (*n*=5) or three (*n*=35) cisplatin administrations, 46 had no delay (11%) and two had a 1-week treatment delay (0.5%). The mean number of cisplatin administrations was 5.3, with a median total cisplatin dose of 420 mg m^−2^ (range, 70–480 mg m^−2^; mean, 379±86 mg m^−2^). The mean duration of treatment was 6.5±1.9 weeks (range, 1–11 weeks; median, 7 weeks). The median achieved dose intensity was 60 mg m^−2^ week^−1^ (range, 10–80 mg m^−2^ week^−1^; mean, 55.7±11.6 mg m^−2^ week^−1^).

Toxicity incidences (scored as the worst CTC grade) are shown in Table 2

**Table 2:** Toxicity (%) of weekly cisplatin in 400 patients (worst toxicity per patient)

**CTC grade^a^**	**0**	**1**	**2**	**3**	**4**
Anaemia	1	34	44	20	1
Leucopenia	10	18	35	30	7
Neutropenia	10	9	25	32	24
Thrombocytopenia	27	34	17	14	8
Nausea	18	44	30	8	0
Vomiting	36	30	27	6	1
Nephrotoxicity	59	32	8	1	0
Hypomagnesaemia	33	52	9	5	1
Hyponatraemia	55	23	18	3	1
Hypokalaemia	81	13	5	1	0
Hypocalcaemia	53	40	5	1	1
Neurotoxicity	53	36	8	3	0
Ototoxicity^b^	58	—	27	14	1

a Common Toxicity Criteria, Version 1.0, National Cancer Institute.

b Audiometry was not routinely performed: grade 0 ototoxicity should be interpreted as grade 0 or 1.

. Nausea and vomiting were prevalent but did not result in dose reduction or cessation of treatment. Reasons not to complete treatment were disease progression (30 patients, 7.5%), haematological toxicity (37 patients, 9%), renal toxicity (29 patients, 7%), ototoxicity (10 patients, 2.5%), neurotoxicity (four patients, 1%), gastrointestinal toxicity (three patients, 1%), cardiovascular complications (two patients, 0.5%), or combinations of reasons including noncompliance and patient's request (22 patients, 5.5%). In total, 12 patients (3%) died within 30 days after the last administration of weekly cisplatin; nine of them had rapidly progressive disease.

### Haematological toxicity

In 37 patients (9%), weekly cisplatin treatment was discontinued because of haematological toxicity, in the majority of them (21 patients) only after the fifth administration. Anaemia was a common adverse event. During the evaluation period 202 patients (51%) received one or more transfusions. The median number of erythrocyte units transfused was two (range, 0–17). Grade 3–4 leucopenia was common, and associated with etoposide cotreatment (OR=2.2, *P*=0.007). Although frequently observed, grade 4 neutropenia was generally brief (<7 days in 74%; <14 days in 94%) and uncomplicated. Febrile neutropenia occurred in only 1.5% of patients. Grade 3–4 thrombocytopenia was observed in 22% of patients who received at least three administrations of weekly cisplatin; 19 patients (5%) received platelet transfusions. Thrombocytopenia was associated with prior chemotherapy (OR=3.2, *P*=0.006) and cisplatin dose (80 mg m^−2^ versus 70 mg m^−2^: OR=2.9, *P*=0.009). Paclitaxel coadministration was not associated with enhanced haematological toxicity (OR=0.39, *P*=0.1 for anaemia; OR=1.9, *P*=0.08 for leucopenia; OR=0.53, *P*=0.2 for neutropenia; OR=0.55, *P*=0.3 for thrombocytopenia).

### Nephrotoxicity

At baseline, serum creatinine (mean±standard deviation) was 84±14 *μ*mol l^−1^ with an estimated creatinine clearance of 83±22 ml min^−1^. After three and six administrations of weekly cisplatin, the serum creatinine was 93±40 and 102±29 *μ*mol l^−1^ respectively with an estimated creatinine clearance of 77±23 and 69±23 ml min^−1^ respectively. A ⩾25% reduction in creatinine clearance was observed in 116 patients (29%). In 164 patients (41%) the serum creatinine rose above the upper limit of normal (grade 1, 127 patients (32%); grade 2, 35 patients (9%); grade 3, two patients (0.5%)). Electrolyte disorders were frequently observed. Mean±standard deviation serum concentrations of magnesium, calcium, sodium and potassium declined from respectively 0.81±0.09 mmol l^−1^ (range, 0.51–1.33 mmol l^−1^), 2.39±0.14 mmol l^−1^ (range, 1.92–3.03 mmol l^−1^), 139±3.7 mmol l^−1^ (range, 128–149 mmoll^−1^) and 4.2±0.41 mmol l^−1^ (range, 2.8–5.5 mmol l^−1^) at baseline to 0.70±0.13 mmol l^−1^ (range, 0.22–1.62 mmol l^−1^), 2.26±0.15 mmol l^−1^ (range, 1.59–2.69 mmol l^−1^), 135±4.3 mmol l^−1^ (range, 117–146 mmol l^−1^) and 4.0±0.50 mmol l^−1^ (range, 2.5–5.2 mmol l^−1^) after three administrations, and 0.62±0.14 mmol l^−1^ (range, 0.23–1.16 mmol l^−1^), 2.24±0.16 mmol l^−1^ (range, 1.53–2.69 mmol l^−1^), 135±4.1 mmol l^−1^ (range, 122–146 mmol l^−1^) and 4.0±0.56 mmol l^−1^ (range, 2.2–5.6 mmol l^−1^) after six administrations of weekly cisplatin.

Results of the logistic regression analysis for nephrotoxicity are shown in Table 3

**Table 3:** Logistic regression analysis for nephrotoxicity

	**Unadjusted**	**Adjusted**^b^		
**Baseline parameter**	**Odds ratio (CI)**	***P*****-value**	**Odds ratio (CI)**	***P*****-value**
*Univariate analysis*				
Age (year^−1^)	1.03 (1.00–1.05)	0.027	1.03 (1.00–1.05)	0.028
Sex (female)	1.71 (1.09–2.70)	0.021	1.46 (0.84–2.57)	0.183
BSA (m^2^)	0.79 (0.24–2.61)	0.696	0.97 (0.28–3.41)	0.966
Performance status >1	1.01 (0.41–2.45)	0.987	0.88 (0.35–2.23)	0.794
Tumour type (ovarian cancer)	1.54 (0.89–2.66)	0.124	0.63 (0.17–2.29)	0.478
Prior carboplatin treatment	0.85 (0.42–1.71)	0.656		
Prior cisplatin treatment	2.16 (1.06–4.39)	0.035		
Cisplatin dose ⩾80 mg m^−2^	1.16 (0.65–2.09)	0.610	2.07 (0.99–4.30)	0.052
Paclitaxel cotreatment	3.46 (1.80–6.66)	<0.001		
Etoposide cotreatment	1.11 (0.66–1.88)	0.687		
Weight loss >5%	0.99 (0.61–1.62)	0.978	1.18 (0.70–1.99)	0.530
Smoking	1.21 (0.75–1.97)	0.436	1.80 (1.01–3.22)	0.046
Alcohol intake >2 units day^−1^	1.25 (0.76–2.03)	0.370	1.77 (0.96–3.23)	0.066
Systolic blood pressure >150 mmHg	1.44 (0.82–2.52)	0.206	1.30 (0.72–2.34)	0.387
Diastolic blood pressure >90 mmHg	0.68 (0.34–1.38)	0.287	0.70 (0.34–1.46)	0.347
Creatinine clearance <70 ml min^−1^	1.24 (0.77–2.01)	0.378	1.04 (0.62–1.74)	0.876
Hyponatraemia (<135 mmol l^−1^)	0.99 (0.50–1.96)	0.968	1.17 (0.58–2.39)	0.657
Hypokalaemia (<4.0 mmol l^−1^)	1.36 (0.79–2.33)	0.271	1.33 (0.76–2.33)	0.319
Hypocalcaemia (<2.2 mmol l^−1^)	0.95 (0.39–2.29)	0.907	0.95 (0.38–2.36)	0.911
Hypomagnesaemia (<0.7 mmol l^−1^)	2.15 (0.80–5.79)	0.129	2.31 (0.82–6.51)	0.113
Anaemia (haemoglobin <normal)	0.95 (0.61–1.49)	0.826	1.11 (0.69–1.79)	0.665
Hypoalbuminaemia (<35 g l^−1^)	3.13 (1.36–7.22)	0.007	3.22 (1.36–7.61)	0.008
Alkaline phosphatase >normal	1.85 (0.93–3.65)	0.078	2.42 (1.17–4.97)	0.016
AST >normal	1.61 (0.65–3.99)	0.308	1.47 (0.57–3.83)	0.428
ALT >normal	1.83 (0.91–3.67)	0.091	1.72 (0.83–3.58)	0.145
LDH >normal	1.10 (0.64–1.89)	0.734	1.19 (0.66–2.16)	0.568
				
				
**Independent risk factor**	**Odds ratio (CI)**	***P*****-value**		
*Multivariate analysis*				
Paclitaxel co-treatment	4.01 (1.83–8.77)	0.001		
Smoking	2.50 (1.39–4.51)	0.002		
Hypoalbuminaemia	3.49 (1.44–8.45)	0.006		
Age (year^−1^)	1.03 (1.01–1.06)	0.007		
Female gender	1.99 (1.09–3.63)	0.025		

a Nephrotoxicity defined as ⩾25% decline in estimated creatinine clearance (Cockroft-Gault).

b Adjusted for prior chemotherapy and cytotoxic cotreatment (paclitaxel, etoposide). CI=95% confidence interval; BSA=body-surface area; AST=aspartate aminotransferase; ALT=alanine aminotransferase; LDH=lactate dehydrogenase.

. In the univariate analysis, age, female sex, prior cisplatin treatment, paclitaxel cotreatment and hypoalbuminaemia were associated with nephrotoxicity (defined as a ⩾25% decline of the estimated creatinine clearance at any time during the evaluation period). After adjustment for prior chemotherapy and additional chemotherapeutical agents, age and hypoalbuminaemia remained significant whereas smoking and elevated serum alkaline phosphatase concentrations were introduced as additional risk factors. The multivariate analysis selected age, female gender, smoking, paclitaxel coadministration and hypoalbuminaemia as independent risk factors. Paclitaxel cotreatment (OR=4.0, *P*=0.001), hypoalbuminaemia (OR=3.5, *P*=0.006) and smoking (OR=2.5, *P*=0.002) were strong predisposing factors for renal toxicity in the multivariate model. There was a gradual increase in renal toxicity with increasing age at an OR of 1.03 year^−1^ (*P*=0.007). Patients younger than 48 years had a 26% risk for renal toxicity, which increased to 35% for patients aged 48–62 years and 41% for patients >62 years. Compared with men, women had a two-fold risk of renal toxicity (OR=2.0, *P*=0.02).

### Neurotoxicity

Clinical data on neurotoxicity (according to CTC criteria) were fully available for the period of weekly cisplatin treatment, but were missing in 71 of our patients (18%) at 2–4 months post-treatment. Furthermore, it is noteworthy that 43 ovarian cancer patients treated with weekly cisplatin in combination with paclitaxel (11% of the study population) received additional 3-weekly treatment with cisplatin and/or paclitaxel immediately following the weekly regimen. Neurotoxicity (mostly peripheral sensory polyneuropathy) was observed in 188 patients (47%) and was mild to moderate in most cases: 145 patients (36%) developed grade 1 neurotoxicity, 33 patients (8%) grade 2, nine patients (2%) grade 3, and one patient experienced grade 4 neurotoxicity.

After univariate analysis a large number of baseline parameters were found to be related with the development of grade 2–4 neurotoxicity: female sex, tumour type (ovarian cancer), prior chemotherapy, cisplatin dose, paclitaxel coadministration, non-smoking and alcohol consumption ⩽2 unitsday^−1^ (Table 4

**Table 4:** Logistic regression analysis for neurotoxicity

	**Unadjusted**	**Adjusted^b^**		
**Baseline parameter**	**Odds ratio (CI)**	***P*****-value**	**Odds ratio (CI)**	***P*****-value**
*Univariate analysis*				
Age (year^−1^)	1.03 (0.99–1.07)	0.129	1.02 (0.98–1.06)	0.286
Sex (female)	4.73 (2.01–11.2)	<0.001	1.53 (0.50–4.65)	0.452
BSA (m^−2^)	0.48 (0.07–3.12)	0.442	0.68 (0.06–7.41)	0.750
Performance status >1	1.42 (0.40–5.11)	0.589	1.23 (0.29–5.22)	0.780
Tumour type (ovarian cancer)	8.69 (3.19–23.6)	<0.001	4.57 (0.63–33.2)	0.133
Prior carboplatin treatment	4.07 (1.71–9.68)	0.001		
Prior cisplatin treatment	6.48 (2.63–16.0)	<0.001		
Cisplatin dose ⩾80 mg m^−2^	0.13 (0.02–0.96)	0.045	0.37 (0.04–3.38)	0.375
Paclitaxel cotreatment	15.3 (4.89–47.9)	<0.001		
Etoposide cotreatment	2.12 (0.67–6.76)	0.203		
Weight loss >5%	0.83 (0.39–1.80)	0.643	2.00 (0.79–5.06)	0.144
Smoking	0.23 (0.09–0.60)	0.003	0.46 (0.16–1.34)	0.153
Alcohol intake >2 units per day	0.27 (0.09–0.79)	0.017	0.42 (0.12–1.43)	0.167
Systolic blood pressure >150 mmHg	1.72 (0.78–3.79)	0.176	1.32 (0.52–3.34)	0.556
Diastolic blood pressure >90 mmHg	0.35 (0.08–1.51)	0.160	0.35 (0.07–1.66)	0.186
Creatinine clearance <70 ml min^−1^	1.72 (0.84–3.53)	0.140	1.07 (0.47–2.45)	0.872
Hyponatraemia (<135 mmol l^−1^)	0.39 (0.09–1.71)	0.214	0.72 (0.15–3.39)	0.678
Hypokalaemia (<3.5 mmol l^−1^)	0.63 (0.24–1.69)	0.361	0.56 (0.19–1.69)	0.303
Hypocalcaemia (<2.2 mmol l^−1^)	1.85 (0.59–5.82)	0.290	2.06 (0.58–7.34)	0.268
Hypomagnesaemia (<0.7 mmol l l^−1^)	0.48 (0.06–3.77)	0.484	0.27 (0.03–2.53)	0.252
Anaemia (haemoglobin <normal)	1.09 (0.54–2.20)	0.801	1.84 (0.80–4.20)	0.149
Hypoalbuminaemia (<35 g l^−1^)	0.82 (0.18–3.69)	0.800	0.77 (0.15–3.96)	0.754
Alkaline phosphatase >normal	0.43 (0.10–1.89)	0.266	0.89 (0.19–4.23)	0.885
AST >normal	1.53 (0.42–5.52)	0.518	1.27 (0.29–5.53)	0.753
ALT >normal	1.37 (0.50–3.79)	0.543	1.08 (0.34–3.47)	0.892
LDH >normal	1.07 (0.46–2.47)	0.871	1.13 (0.43–2.98)	0.809
				
				
**Independent risk factor**	**Odds ratio (CI)**	***P*****-value**		
Paclitaxel coadministration	8.33 (2.43–28.5)	0.001		
Prior cisplatin treatment	3.88 (1.38–10.9)	0.010		
Prior carboplatin treatment	3.50 (1.29–9.48)	0.014		

a Neurotoxicity defined as CTC grade 2–4.

b Adjusted for prior chemotherapy and cytotoxic cotreatment (paclitaxel, etoposide). CI=95% confidence interval; BSA=body-surface area; AST=aspartate aminotransferase; ALT=alanine aminotransferase; LDH=lactate dehydrogenase.

). After adjustment for prior chemotherapy and cytotoxic cotreatment, none of the (other) risk factors remained significant. After multivariate analysis, prior platinum-based chemotherapy (cisplatin or carboplatin) and coadministration of paclitaxel remained independent prognostic indicators for neurotoxicity. The ORs were 8.3 for paclitaxel coadministration (*P*=0.001), 3.9 for pretreatment with cisplatin (*P*=0.01), and 3.5 for pretreatment with carboplatin (*P*=0.01).

### Ototoxicity

Ototoxicity was observed in 168 patients (42%): 110 patients (28%) had CTC grade 2 (reversible tinnitus), 55 (14%) grade 3, and three patients (1%) had grade 4 ototoxicity. Table 5

**Table 5:** Logistic regression analysis for ototoxicity

	**Unadjusted**		**Adjusted^b^**	
**Baseline parameter**	**Odds ratio (CI)**	***P*****-value**	**Odds ratio (CI)**	***P*****-value**
*Univariate analysis*				
Age (year^−1^)	0.99 (0.97–1.02)	0.699	0.99 (0.96–1.02)	0.635
Sex (female)	1.18 (0.65–2.13)	0.595	1.06 (0.51–2.23)	0.871
BSA (m^−2^)	0.25 (0.05–1.28)	0.097	0.31 (0.06–1.65)	0.169
Performance status >1	0.86 (0.24–3.03)	0.815	0.96 (0.27–3.44)	0.945
Tumour type (ovarian cancer)	1.27 (0.63–2.57)	0.501	2.18 (0.43–11.1)	0.347
Prior carboplatin treatment	1.33 (0.60–2.99)	0.484		
Prior cisplatin treatment	0.74 (0.24–2.21)	0.584		
Cisplatin dose ⩾80 mg m^−2^	0.75 (0.32–1.76)	0.506	0.67 (0.25–1.80)	0.430
Paclitaxel cotreatment	1.81 (0.82–4.01)	0.141		
Etoposide cotreatment	0.71 (0.36–1.41)	0.324		
Weight loss >5%	1.34 (0.72–2.50)	0.354	1.54 (0.79–2.97)	0.203
Smoking	1.30 (0.67–2.53)	0.432	1.48 (0.69–3.16)	0.310
Alcohol intake >2 units per day	1.30 (0.69–2.44)	0.413	1.25 (0.59–2.67)	0.561
Systolic blood pressure >150 mmHg	0.65 (0.28–1.51)	0.314	0.66 (0.28–1.60)	0.361
Diastolic blood pressure >90 mmHg	0.36 (0.11–1.20)	0.096	0.40 (0.12–1.36)	0.141
Creatinine clearance <70 ml min^−1^	1.26 (0.67–2.38)	0.479	1.21 (0.62–2.33)	0.577
Hyponatraemia (<135 mmol l^−1^)	0.92 (0.36–2.31)	0.854	1.12 (0.43–2.89)	0.816
Hypokalaemia (<4.0 mmol l^−1^)	0.81 (0.37–1.76)	0.597	0.85 (0.39–1.87)	0.686
Hypocalcaemia (<2.2 mmol l^−1^)	0.82 (0.23–2.87)	0.754	0.93 (0.26–3.32)	0.909
Hypomagnesaemia (<0.7 mmol l^−1^)	1.08 (0.29–3.98)	0.913	0.81 (0.21–3.21)	0.765
Anaemia (haemoglobin <normal)	2.38 (1.25–4.53)	0.008	3.14 (1.57–6.27)	0.001
Hypoalbuminaemia (<35 g l^−1^)	1.96 (0.74–5.25)	0.178	2.38 (0.85–6.66)	0.099
Alkaline phosphatase >normal	0.61 (0.21–1.79)	0.365	0.77 (0.25–2.35)	0.641
AST >normal	1.34 (0.43–4.18)	0.616	1.36 (0.42–4.35)	0.606
ALT >normal	1.31 (0.54–3.17)	0.551	1.41 (0.56–3.53)	0.466
LDH >normal	1.26 (0.63–2.52)	0.508	1.74 (0.81–3.73)	0.159
				
				
**Independent risk factor**	**Odds ratio (CI)**	***P*****-value**		
*Multivariate analysis*				
Anaemia	3.14 (1.57–6.27)	0.001		

a Ototoxicity defined as symptomatic hearing loss (CTCgrade 3–4).

b Adjusted for prior chemotherapy and cytotoxic cotreatment (paclitaxel, etoposide). CI=95% confidence interval; AST=aspartate aminotransferase; ALT=alanine aminotransferase; LDH=lactate dehydrogenase.

shows the results of the logistic regression analysis for ototoxicity defined as symptomatic hearing loss (grade 3–4). Anaemia was the single baseline parameter associated with ototoxicity (OR=3.1, *P*=0.001).

## DISCUSSION

The present study reports the toxic side effects of weekly high-dose cisplatin chemotherapy in 400 patients with locally advanced and/or metastatic cancer. Given the median number of six administrations of weekly cisplatin, the median total dose of 420 mg m^−2^ and the median dose-intensity of 60 mg m^−2^ week^−1^, it can be concluded that a short intensive weekly cisplatin schedule is a feasible treatment option, even in combination with i.v. paclitaxel or oral etoposide.

Haematological toxicity resulted in treatment discontinuation in only 9% of patients (the majority of them only missing one administration of weekly cisplatin). Anaemia, however, was frequently observed, and 51% of the patients received erythrocyte transfusions. Grade 3–4 neutropenia and thrombocytopenia were present, but generally of brief duration and without serious complications. It is noteworthy that paclitaxel cotreatment (in contrast to etoposide coadministration) did not result in enhanced haematological toxicity; this could be explained by a favourable pharmacological interaction between cisplatin and cremophor EL (the vehicle for i.v. paclitaxel administration). It is already known that the sequence paclitaxel–cisplatin induces less profound neutropenia than the alternate sequence, which was first ascribed to lower paclitaxel clearance rates after cisplatin administration (Rowinsky *et al*, 1991). In other studies, however, no pharmacokinetic interaction between paclitaxel and cisplatin could be found (Gelderblom *et al*, 2002), whereas *in vitro* drug accumulation studies have demonstrated significant reduction of intracellular cisplatin concentrations in leucocytes (but not in tumour cells) in the presence of cremophor EL, which was not observed with paclitaxel alone (de Vos *et al*, 1997). The infusion of cremophor EL immediately before cisplatin administration ameliorated leucopenia, neutropenia and thrombocytopenia (Gelderblom *et al*, 2002), which may be of potential interest for improvement of the therapeutic index of weekly cisplatin treatment.

Renal toxicity was present and necessitated discontinuation of weekly cisplatin treatment in 7% of patients. According to CTC criteria, nephrotoxicity was observed in 42% of patients (serum creatinine above the upper limit of the normal); the majority of them (32%) experienced mild (grade 1) renal toxicity, whereas 5% of the patients already had elevated serum creatinine concentrations at baseline. The estimated creatinine clearance declined from 83±22 ml min^−1^ at baseline to 69±23 ml min^−1^ after six administrations of weekly cisplatin. In 116 patients (29%), creatinine clearance decreased 25% or more; the median decrease in creatinine clearance was 16%. This certainly does not exceed the nephrotoxicity reported from conventional 3-weekly cisplatin treatment, and confirms previous observations that haematological, and not renal, toxicity is the major dose-limiting adverse event of weekly high-dose cisplatin chemotherapy (Planting *et al*, 1993,1997a,1997b). The administration of cisplatin in a solution with hypertonic saline may have alleviated renal toxicity, thus allowing dose-dense cisplatin treatment. In animal models it has been shown that administration of cisplatin in a vehicle of hypertonic saline remarkably reduced nephrotoxicity without loss of antitumour activity (Litterst, 1981). The most likely explanation is that chloride excess results in the decreased formation of highly nephrotoxic hydrolysis products of cisplatin (Earhart *et al*, 1983; Bajorin *et al*, 1986; Jones *et al*, 1991).

Several baseline parameters were identified as independent prognostic indicators for renal toxicity. The incidence of nephrotoxicity gradually increased with age (OR=1.03 year^−1^). Although increased age was a risk factor for nephrotoxicity, our study also demonstrates that weekly cisplatin treatment is not necessarily contraindicated in elderly patients. Women had a two-fold increased risk for renal toxicity compared with men. The reason for this gender difference is not known. In a previous study (de Jongh *et al*, 2001), we found that unbound cisplatin clearance was 15% higher in men than in women but age had no significant influence on this clearance. Paclitaxel coadministration was strongly related to the development of nephrotoxicity (OR=4.0, CI=1.8–8.8). Although the mechanism of this association is not clear, it is in concordance with a report of increased nephrotoxicity with paclitaxel/cisplatin combination as compared to cisplatin single-agent chemotherapy in a small group of patients with gynaecologic cancers (Merouani *et al*, 1997). Smoking also was an independent risk factor for cisplatin-induced nephrotoxicity in the present study (OR=2.5, CI=1.4–4.5). To our knowledge, this has not been reported in the literature, and the underlying pathophysiological mechanism remains a matter of speculation. It is known, however, that cigarette smoking is associated with oxidative stress (Maytin *et al*, 1999), which could possibly lead to enhanced formation of nephrotoxic platinum metabolites. Although it cannot be excluded that smoking was associated with nephrotoxicity through coexisting smoking-related cardiovascular disease, other indicators for cardiovascular disease such as hypertension and diminished baseline creatinine clearance were not identified as risk factors for cisplatin-induced nephrotoxicity. Furthermore, there was no association between nephrotoxicity and a history of hypertension, cardiovascular disease or diabetes mellitus in 425 patients treated with conventional cisplatin chemotherapy (Stewart *et al*, 1997). Another strong predisposing factor for renal toxicity was hypoalbuminaemia. This has also been described for patients receiving conventional cisplatin treatment (Stewart *et al*, 1997). Various studies have demonstrated that cisplatin-induced nephrotoxicity is related to the peak plasma concentration and/or the area under the plasma concentration–time curve of nonprotein bound cisplatin (Reece *et al*, 1987; Nagai *et al*, 1996; Nagai and Ogata, 1997). It is postulated that low serum albumin concentrations are associated with increased plasma concentrations of unbound cisplatin, resulting in enhanced renal toxicity. It is noteworthy that cisplatin dose (in the range 70–80 mg m^−2^) was not associated with nephrotoxicity and that baseline creatinine clearance did not predict for nephrotoxicity (defined as relative decrease in estimated creatinine clearance), both findings confirming data on conventional cisplatin treatment (Lagrange *et al*, 1997).

Neurotoxicity was found to be acceptable with weekly cisplatin chemotherapy. According to previous studies, neurotoxicity is mainly related to cumulative cisplatin dose, and shortening of the treatment interval does not necessarily lead to worsening of the neurotoxic side effects (Cavaletti *et al*, 1992; Hilkens *et al*, 1994,^1995^). Neurotoxicity was evaluated during and immediately following the weekly cisplatin regimen. Since cisplatin-induced neuropathy can worsen during the first months after cisplatin treatment (Hilkens *et al*, 1994), it was also assessed 2–4 months after the completion of the weekly cisplatin regimen. The worst toxicity score was used for evaluation of neurotoxicity. Here, several biases were met. First, data on neurotoxicity at 2–4 months were not traceable in 71 patients (18%), which probably led to some underestimation of neurotoxicity. On the other hand, 11% of the total study population received additional paclitaxel and/or cisplatin immediately following the weekly cisplatin regimen with possible overestimation of neurotoxicity. Nevertheless, only four patients (1%) did not complete treatment due to neurotoxicity. CTC grade 2–4 neurotoxicity was observed in 11% of the patients.

A large number of baseline parameters were identified as potential risk factors for cisplatin-induced neurotoxicity by univariate logistic regression analysis (Table 4): female sex, ovarian cancer, prior platinum-based chemotherapy, individual cisplatin dose, nonsmoking and alcohol consumption <3 units daily. After adjustment for prior chemotherapy and cotreatment (paclitaxel, etoposide), all other baseline parameters were eliminated as risk factors. In the multivariate model, paclitaxel coadministration and prior cisplatin and/or carboplatin treatment were identified as independent risk factors for grade 2–4 neurotoxicity. The found associations could be anticipated and this unfortunately does not add much to our knowledge of cisplatin-induced neuropathy. Both paclitaxel and cisplatin are neurotoxic agents, and a combination of cisplatin with taxanes is known to result in increased neurotoxicity (Connelly *et al*, 1996; Hilkens *et al*, 1997). Furthermore, cisplatin-induced neurotoxicity is mainly dependent on the cumulative cisplatin dose (Cavaletti *et al*, 1992; Hilkens *et al*, 1994,^1995^). However, despite an increased risk of neurotoxicity in the platinum pretreated patients, severe neurotoxicity necessitating treatment discontinuation rarely occurred. This is in concordance with a previous study demonstrating that patients with absent or mild signs of neuropathy after prior treatment with cisplatin to a cumulative dose of 400–450 mg m^−2^ can be retreated with six cycles of cisplatin 50–70 mg m^−2^ weekly with only a minimal risk of significant neurotoxicity, not different from that in carboplatin pretreated patients (van den Bent *et al*, 2002).

Ototoxicity is another major side effect of cisplatin chemotherapy, and is probably caused by cisplatin-induced degeneration of the hair cells of the cochlea. Previous studies have shown that ototoxicity is related to both cumulative and individual cisplatin dose (Reddel *et al*, 1982; Schaeffer *et al*, 1985; Laurell and Jungnelius, 1990). In the present study, tinnitus occurred in 25% of the patients, 15% had subjective, symptomatic hearing loss, and in 2.5% weekly cisplatin treatment was not completed due to ototoxicity.

Anaemia was a predisposing factor for grade 3–4 ototoxicity (OR=3.1, CI=1.6–6.3). The pathophysiological background of this association is presently unknown. Others previously identified anaemia as a risk factor for cisplatin-induced ototoxicity (Blakley *et al*, 1994). They also found a relation with hypoalbuminaemia, which was a borderline prognostic factor (OR=2.4, CI=0.9–6.7, *P*=0.1) in the present study on weekly cisplatin. It is noteworthy that age, sex, performance status, creatinine clearance and individual cisplatin dose (in the range 70–80 mg m^−2^) were not associated with ototoxicity. Remarkably, in the present analysis, performance status was not associated with any chemotherapy-induced toxicity, but this is probably related to the selection of patients with good performance status to treat with dose-dense cisplatin chemotherapy.

An advantage of the weekly regimen is shortening of the treatment period from 18–24 weeks with standard treatment (six courses with intervals of 3–4 weeks) to 6–8 weeks with weekly treatment using similar total cisplatin dose. On theoretical grounds it can be expected that weekly cisplatin treatment enhances antitumour activity. Indeed, weekly cisplatin in combination with either etoposide or paclitaxel was highly active and well tolerated in the patients with advanced ovarian cancer, and even in the case of platinum-refractory disease (defined as platinum treatment-free interval <4 months) the objective response rate was in the order of 50% (van der Burg *et al*, 1998,^2002^). This suggests that platinum resistance is a relative phenomenon that could be overcome by shortening the treatment interval and supports the use of weekly platinum treatment in patients with relapsed ovarian cancer. For other tumour types, however, the place of weekly cisplatin treatment remains to be determined.

In conclusion, in a large cohort of patients, we have demonstrated that weekly cisplatin at doses of 70–80 mg m^−2^ administered in hypertonic saline is a feasible treatment option, even when combined with oral etoposide or i.v. paclitaxel. Predisposing factors for treatment-related toxicity differ from side effect to side effect.
